# Targeting ferroptosis in sepsis-induced cardiomyopathy: mechanisms and therapeutic potential of traditional Chinese Medicine

**DOI:** 10.3389/fphar.2026.1785167

**Published:** 2026-08-21

**Authors:** Guoxia Zhang, Hongshuan Liu, Qing Zhang, Shuli Cheng, Shuce Ling, Jingwen Xue, Nan Kou, Jingqin Wu, Xinyuan Li, Hao Yang, Fan Yuan, Yanwei Xing, Nan Guo

**Affiliations:** 1 Dongzhimen Hospital, Beijing University of Chinese Medicine, Beijing, China; 2 Beijing University of Chinese Medicine, Beijing, China; 3 Guang’anmen Hospital, China Academy of Chinese Medical Sciences, Beijing, China

**Keywords:** ferroptosis, iron, lipid peroxidation, sepsis-induced cardiomyopathy, therapeutic target, traditional Chinese medicine

## Abstract

Sepsis-induced cardiomyopathy (SIC), a severe complication of sepsis, is strongly associated with significantly increased patient mortality, yet targeted therapeutic strategies remain lacking. Emerging evidence has established ferroptosis, an iron-dependent, lipid peroxidation-mediated regulated cell death pathway, as a critical pathogenic mechanism underlying SIC. Iron metabolism dysregulation plays a central role in SIC. Although sepsis reduces circulating iron levels, iron paradoxically accumulates in myocardial tissues. This abnormal redistribution of iron leads to uncontrollable lipid peroxidation in cardiac tissue, ultimately aggravating myocardial injury. In recent years, the application of traditional Chinese medicine (TCM) in the treatment of sepsis has become increasingly widespread. Moreover, due to its multi-target and multi-pathway advantages, TCM has shown considerable potential in the intervention of ferroptosis. However, due to the lack of understanding of the precise molecular mechanism, the therapeutic potential of TCM and its active components in SIC has not been fully explored. This review summarizes the mechanism of ferroptosis in the pathogenesis of SIC, and comprehensively synthesizes the mechanism and application potential of TCM targeting ferroptosis for SIC intervention, aiming to provide a theoretical basis for the development of innovative treatment strategies and the improvement of prognosis of SIC.

## 1 Introduction

Sepsis is defined as life-threatening organ dysfunction triggered by dysregulated host responses to infection, which remains a major threat to human health (Singer et al., 2016; Lelubre and Vincent, 2018). Epidemiological data from 2017 showed that there were 48.9 million newly diagnosed sepsis cases and 11 million sepsis-associated deaths globally, with the latter accounting for 19.7% of all deaths worldwide (Meyer and Prescott, 2024; Rudd et al., 2020). The incidence of sepsis-induced cardiomyopathy (SIC) ranges from 40% to 60% within 3 days following sepsis diagnosis (Aneman and Vieillard-Baron, 2016). SIC is defined as reversible myocardial dysfunction without underlying heart disease during sepsis (Martin et al., 2019). SIC can increase the mortality rate of patients with sepsis. The results of a 50-year epidemiological study show that the mortality rate of patients with SIC is 3.5 times that of patients with simple sepsis (Rabuel and Mebazaa, 2006), and the mortality rate of sepsis patients combined with SIC is as high as 70% (Singer et al., 2016). Therefore, SIC is an important predictor of sepsis mortality. SIC seriously affects the prognosis of patients with sepsis. Although the concept of SIC proposed firstly by Parker et al. over 40 years ago, the core treatment of SIC at present still lies in the early identification of sepsis, optimization of hemodynamics, and active correction of risk factors. There is no targeted treatment yet (Evans et al., 2021; Hotchkiss and Karl, 2003). Moreover, there are various types of cardiac dysfunction, including systolic dysfunction (which can manifest as low or high motility) and/or diastolic dysfunction, which can occur alone or simultaneously. The treatment of SIC requires individualized management based on precisely identified cardiovascular phenotypes, and there is no unified treatment guideline (Aissaoui et al., 2025; Hollenberg and Singer, 2021). The mechanism of SIC is very complex, involving inflammatory responses, oxidative stress, coagulation dysfunction, and mitochondrial dysfunction, *etc.* (Rabuel and Mebazaa, 2006; Evans, 2021).

Recent studies have further clarified and emphasized that ferroptosis, as an iron-dependent cell death driven by lipid peroxidation, is the core pathological process mediating the occurrence and development of SIC (Fang et al., 2021; Li et al., 2020). Ferroptosis specific inhibitor ferrostatin-1 (Fer-1) has been shown to effectively alleviate SIC (Xiao et al., 2021; Van Coillie et al., 2022). Based on the GSE79962 dataset, which contains human myocardial tissue samples from 20 SIC patients and 11 healthy controls (platform: GPL6244), Song et al. identified core genes in SIC by integrating differential expression analysis, WGCNA, and cuproptosis-related and ferroptosis-related gene sets. The study first screened 173 differentially expressed genes using a threshold of adjusted *P* < 0.05 and |log_2(fold change)| > 1). Subsequently, WGCNA was performed with a soft threshold β = 5, scale independence R^2^ = 0.87, combined with gene significance >0.2 and module membership >0.8, identifying 411 hub module genes. Meanwhile, ferroptosis- and cuproptosis-related genes were obtained from the FerrDb V2 database and the literature, respectively. By taking the intersection of these three sets, three core genes—cytochrome P450 oxidoreductase, solute carrier family 7 member 5, signal sensor and transcription activator 3—were ultimately identified. Their diagnostic value was validated by ROC curve analysis, with area under the curve values of 0.9227, 0.9909, and 0.9636, respectively (Song J. et al., 2023). Based on GSE185754 and GSE171546, both derived from C57BL/6J mouse myocardial tissues (platform: GPL24247, Illumina NovaSeq 6000), fuzzy clustering identified genes like heme oxygenase 1 (HO-1) and solute carrier family 7 member 11 (SlC7A11), whose expression aligns with ferroptosis progression (Xu and Bu, 2023). Human septic heart transcriptome analysis confirmed widespread ferroptosis-related genes dysregulation. Hub genes hypoxia inducible factor 1 subunit alpha (HIF1-α), NADPH oxidase 4 and tumor protein p53 (P53) demonstrated diagnostic and prognostic utility (Zou et al., 2023). Machine learning and animal studies validated ribonucleotide reductase regulatory subunit M2 as a ferroptosis-related key gene, suppressed by Fer-1 to improve sepsis outcomes (He, 2024). In addition, ferroptosis inhibitor attenuate SIC *in vitro* and *in vivo* (Wang et al., 2020; Li et al., 2020). Taken together, these results highlight ferroptosis as a pivotal, dynamically modulated mechanism in SIC, positioning ferroptosis as promising diagnostic and therapeutic targets.

Ferroptosis is a unique type of regulated cell death that was first defined in 2012 (Dixon et al., 2012). This iron-dependent cell death pathway is characterized by the uncontrollable accumulation of lipid peroxides, causing oxidative damage to cellular membranes and plasma membranes (Stockwell, 2022). It occurs due to the failure of antioxidant defenses, such as glutathione peroxidase 4 (GPX4), which normally detoxify lipid hydroperoxides in the cell membrane, ultimately producing too much lipid peroxide (Yang et al., 2014). Iron is a key driving factor for the occurrence and development of ferroptosis. Iron regulates ferroptosis through two mechanisms. Firstly, Fe^2+^ can catalyze H_2_O_2_ to generate hydroxyl radicals (·OH) through the Fenton reaction, which can attack polyunsaturated fatty acids (PUFAs) on the cell membrane and trigger lipid peroxidation (Dixon, 2014). Lipid peroxidation products like malondialdehyde (MDA) and 4-hydroxynonenal (4-HNE) can impair the structural integrity of cell membranes and lead to the loss of cell function. If iron is not effectively stored or utilized after entering the cell through the transferrin receptor (TFR1), the abnormal accumulation of Fe^2+^ will further accelerate the Fenton reaction process (Noguchi et al., 2025). Secondly, some lipid peroxidases, such as lipoxygenases, rely on iron as a cofactor to efficiently promote the oxidation of PUFAs, thereby aggravating the lipid peroxidation cascade (Stoyanovsky, 2019). When the antioxidant system fails to timely clear these lipid peroxides, iron-dependent enzymatic activity further exacerbates oxidative damage. In addition, when the intracellular antioxidant system, especially GPX4 and glutathione (GSH), fails to effectively remove lipid peroxidation products, eventually leading to uncontrolled lipid peroxidation and promoting the continuous progression of ferroptosis (Stockwell, 2017).

Increasing studies have demonstrated the potential of Traditional Chinese Medicine (TCM) in sepsis treatment. Systematic review showed that TCM can reduce sepsis-induced organ dysfunction through various mechanisms (Song Y. et al., 2023). Chinese herbal medicine can play a protective role by enhancing antioxidant capacity and regulating ferroptosis related signaling pathways (He et al., 2025; Jiang et al., 2019). Therefore, this review elaborates on the pathological mechanisms underlying ferroptosis in SIC in detail, while summarizing the research advances regarding TCM targeting ferroptosis for SIC treatment, aiming to provide a theoretical basis for the development of treatment strategies and thereby improve the clinical prognosis of SIC patients.

## 2 Literature search strategy

This narrative study aims to conduct a flexible, comprehensive and explanatory academic integration in the field of “Traditional Chinese Medicine regulating ferroptosis for the treatment of sepsis-induced cardiomyopathy”. It did not follow the PRISMA guidelines required for systematic reviews for literature screening and quality assessment. We searched three databases: PubMed, Web of Science and China National Knowledge Infrastructure (CNKI). The search time range was from January 2012 to December 2025, as the concept of ferroptosis was only proposed in 2012. The search keywords were combined using both subject terms and free words. The core structure of the search strategy is as follows: sepsis AND (heart OR cardiac OR cardio* OR myocard*) AND (ferroptosis OR iron OR “lipid peroxidation”). Relevant studies related to TCM or Chinese herbal medicine were then selected through screening of titles, abstracts, and full texts. The selection was mainly based on the following considerations: (1) The research type was an original research study, including *in vitro* experiments and/or *in vivo* experiments; (2) The intervention measures were a single Chinese herbal component or a Chinese herbal formula; (3) The outcome indicators clearly involved ferroptosis-related indicators and cardioprotective effects, such as GPX4, acyl-CoA synthetase long chain family member 4 (ACSL4), MDA, GSH, Fe^2+^, etc; (4) All included studies were published in peer-reviewed journals, with complete experimental designs and clear data reporting.

## 3 Overview of iron metabolism disorder in sepsis

Systemic iron dysregulation is a characteristic pathological feature of sepsis, with the core manifestation being early hypoferremia, and this pathological change has a significant impact on clinical prognosis. Clinical data show that within 24–72 h after sepsis patients are admitted to the ICU, the incidence of hypoferremia and inflammatory anemia (AI) in patients with sepsis can reach 60%–80% (Jiang et al., 2019). There were significant changes in iron metabolism-related indicators, including significant increases in hepcidin and ferritin levels, accompanied by decreases in serum iron, transferrin saturation and erythrocyte ferritin levels. Moreover, the changes in the above indicators often occur earlier than the obvious AI (Boshuizen et al., 2018). It is worth noting that these iron metabolism indicators have prognostic value. Among them, the predictive specificity of elevated hepcidin levels for the 28-day mortality rate of patients with sepsis was 87.3%, which was better than the traditional clinical evaluation index of the sequential organ failure assessment (SOFA) score (Jiang et al., 2019). A prospective study of 61 sepsis patients further validated the prognostic significance of iron parameters, reporting that increased serum iron, ferritin, and transferrin saturation and decreased transferrin were associated with the SOFA score, mortality, and reduced survival (Brandtner, 2020).

During acute infection, hepcidin levels rise rapidly, whereas iron levels fall, dropping below the normal range in 95.8% of cases. Later, hepcidin significantly decrease during the recovery stage, and the iron concentration that had dropped recovered. In the case of persistent inflammation, iron levels remain low (Moro et al., 2023). Iron overload increases susceptibility to infection by promoting pathogen growth and weakening immunity. A cohort study of 142,188 found that in the general population, genetic iron overload (C282Y homozygous) can significantly increase the risk of infection. Each 1 μmol/L increase in serum iron was associated with a 2% increase in overall infection risk and a 47% increase in sepsis risk in individuals homozygous for C282Y in a dose-dependent manner (Mottelson et al., 2024). This hepcidin-mediated hypoferremia constitutes a host defense mechanism by restricting iron availability for pathogen proliferation. Crucially, hepcidin deficiency exacerbates sepsis mortality (73.9% vs. 46.7% in hepatic knockout mice), whereas iron restriction rescues survival (Zeng et al., 2015). Besides, airway-derived hepcidin demonstrates lung-protective effects (Chen et al., 2014).

Admission iron parameters strongly correlate with survival: low serum iron (≤5.8 μmol/L), low transferrin saturation (≤15%), and high transferrin (>1.6 g/L) are associated with improved short-term and long-term prognosis (Tacke et al., 2016). Cohort studies further validate the clinical stratification utility of hepcidin and ferritin: ferritin levels in septic shock exceed sterile inflammation by > 4-fold, while the hepcidin-to-ferritin ratio predicts septic shock risk (AUC = 0.79) (Hortová-Kohoutkov et al., 2023).

Critically, iron redistribution creates a pathogenic dichotomy: while serum hypoferremia limits microbial growth, intracellular iron accumulation (particularly in cardiomyocytes) promotes reactive oxygen species (ROS) production *via* the Fenton reaction, precipitating oxidative damage and organ dysfunction–establishing a direct mechanistic link to ferroptosis. This process precipitates oxidative damage and organ dysfunction, thereby establishing a direct mechanistic connection to ferroptosis.

## 4 Mechanisms of ferroptosis in sepsis-induced cardiomyopathy

### 4.1 Iron metabolism dysregulation: a core trigger of cardiomyocyte ferroptosis

**FIGURE 1:**
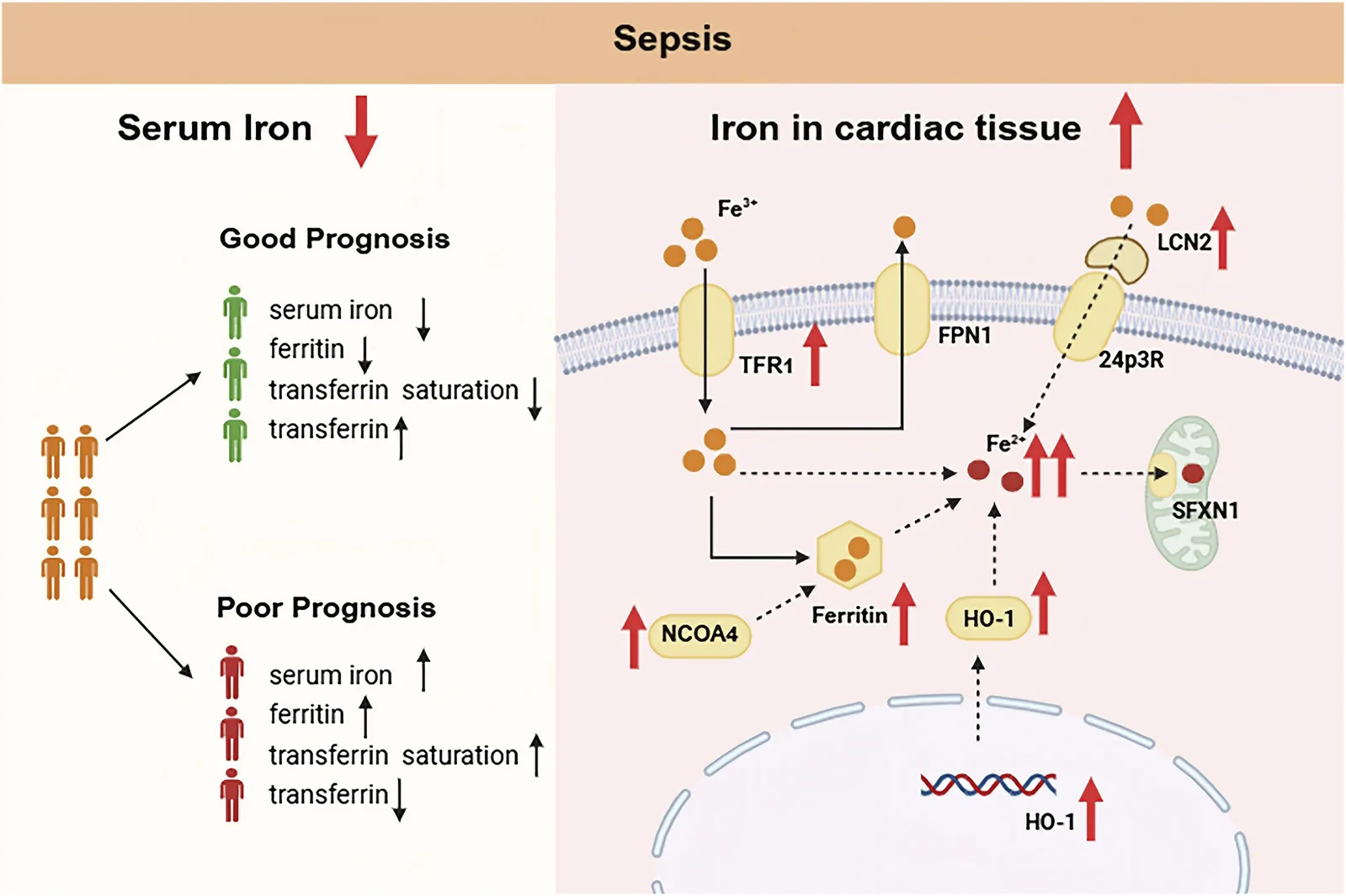
Cardiac Iron Overload and Serum Iron Deficiency in Sepsis-Induced Cardiomyopathy. In sepsis patients, iron metabolism related indicators have prognostic value: decreased serum iron level, ferritin, transferrin saturation and increased transferrin level suggest a good prognosis. In sepsis, serum iron is reduced while iron accumulates in the heart tissue. Mechanistically, the upregulation of TFR1 expression leads to excessive iron intake in cardiomyocytes. Elevated expression of NCOA4 promotes an increase in intracellular iron release. The upregulation of HO-1 expression accelerates the degradation of heme and releases a large amount of iron. The LCN2/24p3R complex can carry iron and be internalized into the cytoplasm and subsequently increase intracellular iron. These factors ultimately work together to mediate iron accumulation in cardiac tissue. TFR1: transferrin receptor; FPN1: ferroportin 1; SFXN1: sideroflexin 1; NCOA4: nuclear receptor coactivator 4; HO-1: heme oxygenase-1; LCN2: lipocalin 2.

Although circulating iron is reduced in sepsis, iron accumulates in myocardial tissue (Figure 1). The researchers found that in the heat-killed *Escherichia coli* ^+/−^ induced sepsis model, co-injection of iron sucrose approximately doubled the amount of TNF-α (1400 vs. 2600 pg/mL), which greatly upregulated HO-1 mRNA and protein expression. It caused oxidative stress damage to cardiac tissue and greatly increased the mortality rate of mice (Ra et al., 2004). Iron chelators such as Fer-1 and dexrazoxane can alleviate cardiac damage in sepsis, characterized by reduced creatine kinase MB isoenzymes (CK-MB), lactate dehydrogenase, and cardiac troponin I (cTnI), as well as improved systolic function in cardiac ultrasound, validating iron overload as a therapeutic target (Li et al., 2020).

Iron homeostasis disruption constitutes the foundational event driving ferroptosis in SIC. In sepsis, intracellular iron accumulation is orchestrated by multiple pathways. Lipopolysaccharide (LPS) upregulates nuclear receptor coactivator 4 (NCOA4), which interacts with ferritin and facilitates its degradation through ferritinophagy, thereby releasing substantial amounts of labile Fe^2+^ into the cytoplasm. It is worth noting that the labile iron pool (LIP) refers to the collection of Fe^2+^ with redox activity within the cell, which can directly participate in the catalytic generation of ROS in the Fenton reaction and is the key executor of ferroptosis (Zhang, 2024). The study by Huang et al. further revealed that LPS can induce the recruitment of peripheral neutrophils to the cardiac tissue and subsequently release lipocalin 2 (LCN2). LCN2 bound to receptor 24p3R and transported iron to cardiomyocytes, thereby increasing LIP and causing ferroptosis (Huang et al., 2022). Regarding other iron transport proteins, one study has shown that LPS leads to an upregulation of TFR1 and Ferritin, but this study did not conduct any related rescue experiments (Chen et al., 2022).

Mitochondria serve as the primary production centers for cellular ATP, a role that is foundational to cardiac function (Xu et al., 2025; Hinton, 2024). In sepsis, mitochondria are dysfunctional and their energy production capacity is significantly reduced, which is the direct cause of reduced myocardial contractility (Rudiger and Singer, 2007). The pathogenic association between mitochondrial damage and SIC has been fully confirmed (Hu, 2024). Recent studies have further confirmed that ferroptosis is involved in mitochondrial dysfunction (Ye et al., 2024). Mechanistically, excess Fe^2+^ in the cytoplasm activated the mitochondrial membrane-specific sideroflexin 1, which transported Fe^2+^ to the mitochondrial matrix, leading to the generation of large amounts of mitochondrial ROS and initiating the lipid peroxidation cascade (Li et al., 2020).

### 4.2 Lipid peroxidation and oxidative stress: executors of ferroptosis

**FIGURE 2:**
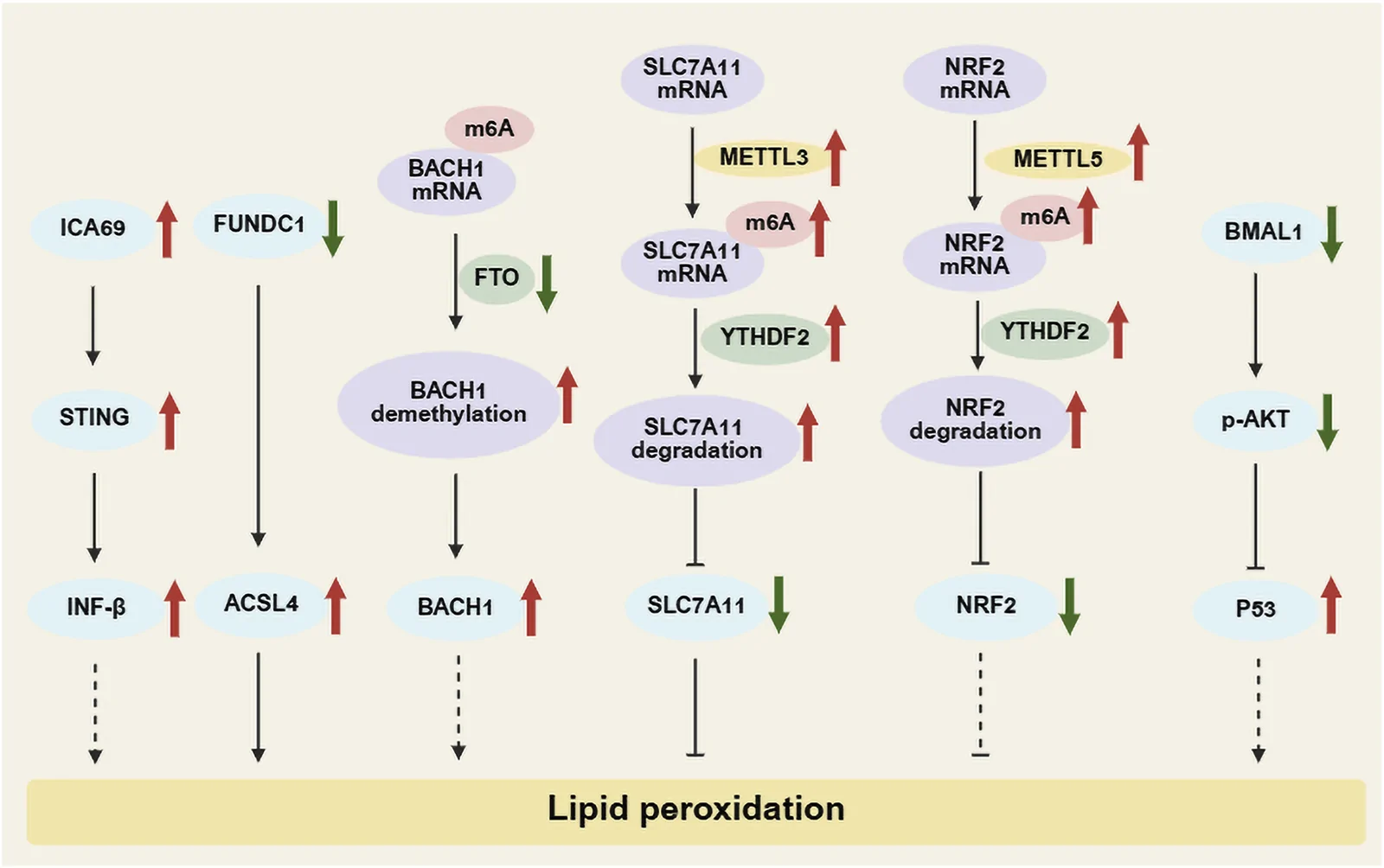
Mechanisms of lipid peroxidation in sepsis-induced cardiomyopathy. The abnormal accumulation of lipid peroxidation in cardiac tissue in sepsis is synergistically driven by multiple signaling pathways. The pathological process mainly involves the activation of the ICA-69-STING and FUNDC1-ACSL4 signaling axes, as well as abnormal post-transcriptional regulation mediated by m^6^A RNA modification. The latter specifically includes the accumulation of BACH1 protein due to the downregulation of FTO, the decreased expression of SLC7A11 caused by the upregulation of METTL3/YTHDF2, and the decreased expression of NRF2 due to the upregulation of METTL5/YTHDF2. Furthermore, the abnormal activation of the BMAL1-p-AKT-P53 pathway further exacerbates the imbalance of redox homeostasis. The above-mentioned mechanisms jointly trigger fatal lipid peroxidation reactions and myocardial damage. ICA69: islet cell autoantigen 69; INF-β: interferon beta; FUNDC1: FUN14 domain-containing protein 1; ACSL4: acyl-CoA synthetase long chain family member 4; BACH1: BTB domain and CNC homolog 1; SLC7A11: solute carrier family 7 member 11; METTL3: methyltransferase 3; YTHDF2: YTH N6-methyladenosine RNA binding protein F2; NRF2: nuclear factor erythroid 2-related factor 2; METTL5: methyltransferase 5; BMAL1: basic helix-loop-helix ARNT-like protein 1; p-AKT: phosphorylated protein kinase B; P53: tumor protein p53.

Uncontrolled lipid peroxidation drives the occurrence of ferroptosis in SIC. This peroxidation is primarily fueled by the accumulation of ROS, which is triggered by iron overload and mitochondrial dysfunction. Substantial evidence indicates that sepsis induces the accumulation of lipid peroxides in the heart, as demonstrated by elevated levels of markers such as MDA and 4-HNE in experimental models (Wang et al., 2020; Kong et al., 2022). The process involves an imbalance between the ferroptosis driver and suppressor. The ferroptosis driver, such as prostaglandin-endoperoxide synthase 2 (PTGS2) and ACSL4 is upregulated while the ferroptosis suppressor like GPX4, GSH, and superoxide dismutase (SOD) is downregulated. These alterations are not model-specific but are a consistent feature of SIC, as evidenced in both cecal ligation and puncture (CLP) models (Li et al., 2024) and LPS-induced models (Kong et al., 2022; She et al., 2023). In sepsis, multiple signaling pathways interweave and activate synergistically, promoting lipid peroxidation and ultimately inducing cardiac dysfunction (Figure 2).

#### 4.2.1 ICA69-STING axis

Recent studies have shown that LPS upregulated the expression of islet cell autoantigen 69 (ICA69), thereby activating the interferon-stimulating factor gene (STING). STING activation triggerred intracellular lipid peroxidation, a hallmark event in ferroptosis (Ding et al., 2025; Hu et al., 2022). In septic mouse models, knockout of ICA69 reduced ferroptosis markers such as PTGS2, ROS, and MDA increased anti-lipid peroxidation enzymes such as GPX4 and SOD, while concurrently improving cardiac function, further validating the role of this pathway in SIC-related ferroptosis (Kong et al., 2022).

#### 4.2.2 FUNDC1-ACSL4 axis

Clinical data show that the plasma level of FUN14 domain 1 (FUNDC1) in SIC patients was significantly reduced. Experimental studies have further confirmed that FUNDC1 knockout (FUNDC1^−/−^) can significantly aggravate CLP-induced cardiac dysfunction, specifically manifested as decreased EF%, impaired myocardial contractile function, accompanied by enhanced oxidative stress and ferroptosis. The expression of GPX4 and SLC7A11 was downregulated, and the expression of ACSL4 was significantly increased. Crucially, the co-immunoprecipitation experiment confirmed that FUNDC1 interacted with ACSL4, and FUNDC1 could inhibit ACSL4. Therefore, loss of FUNDC1 mediated lipid peroxidation through activation of ACSL4 (Li et al., 2024).

#### 4.2.3 M6A RNA modification and ferroptosis crosstalk

Increasing evidence has shown that m6A RNA methylation is closely related to ferroptosis in the pathogenesis of sepsis and its complications, including SIC. The core link of the interaction between the two is that sepsis can induce abnormal changes in the modification level of m6A, and then regulate the expression of key ferroptosis-related genes at the post-transcriptional level, ultimately aggravating the process of cellular ferroptosis and organ dysfunction (Wu et al., 2024; Lai et al., 2024). This regulatory process is dynamically mediated by the “Writer” (methyltransferase), “Eraser” (demethylase), and “Reader” (methylated reading protein) family members related to m6A modification. These molecules regulate the stability and translation efficiency of the target mRNA through synergistic action (Sang et al., 2024). The specific mechanistic pathways are shown below.

##### 4.2.3.1 FTO-m6A-BACH1 axis

Fat mass and obesity-associated protein (FTO), an m^6^A demethylase (“eraser”), can participate in gene expression regulation by dynamically regulating RNA methylation levels, thereby influencing the ferroptosis in the progression of SIC. Experimental studies have confirmed that overexpression of FTO has a protective effect on septic hearts, manifested as inhibiting ferroptosis, reducing myocardial inflammatory responses, improving cardiac function impairment, and increasing the survival rate (Sang et al., 2024). The molecular mechanism of this protective effect was closely related to the decreased m^6^A modification level on BTB and CNC homolog 1 (BACH1) mRNA mediated by FTO (Sang et al., 2024; Zeng, 2024). BACH1 is a transcription factor that promotes ferroptosis, driving ferroptosis by inhibiting the expression of antioxidant genes and exacerbating oxidative stress (Su et al., 2023; Namgaladze et al., 2022). The expression of BACH1 was negatively correlated with FTO, and the cardioprotective effect of FTO depended on the downregulation of BACH1. This dependence was confirmed by functional rescue experiments. BACH1 overexpression reversed the FTO-mediated cardioprotective effect (Zeng, 2024).

##### 4.2.3.2 METTL3-YTHDF2-SLC7A11 axis

In contrast to protective erasers like FTO, the m^6^A “writer” methyltransferase 3 (METTL3) exhibits a pro-ferroptotic role in SIC. Research has shown that METTL3 is upregulated in LPS-stimulated cardiomyocytes and directly mediates the m^6^A modification of SLC7A11 mRNA. This modification serves as a recognition signal for the “reader” YTH N6-Methyladenosine RNA Binding Protein F2 (YTHDF2), which binded to the marked transcript and promoted its decay, as confirmed by mechanistic studies (Shen et al., 2023). The loss of SLC7A11, a critical component of the antioxidant defense, disrupted redox homeostasis, leading to excessive lipid peroxidation, thereby inducing ferroptosis.

##### 4.2.3.3 METTL5-YTHDF2-NRF2 axis

Similarly, the role of the methyltransferase 5 (METTL5)-YTHDF2-nuclear factor erythroid 2-related factor 2 (NRF2) axis has also been recognized. METTL5 expression and m6A levels were elevated in sepsis models. METTL5 mediated m6A methylation on the mRNA of the transcription factor NRF2. YTHDF2 bound to the m6A-modified NRF2 mRNA, triggering its degradation. Suppression of NRF2 abolished its protective effect against ferroptosis, resulting in increased lipid ROS, MDA, iron overload, and cardiomyocyte death (Wang et al., 2025).

#### 4.2.4 Circadian rhythm and ferroptosis crosstalk

The results of a large cohort study involving 409,570 people with an average follow-up period of 13.54 years showed that a healthy sleep pattern could reduce the risk of sepsis. Each 1-point increase in sleep score was associated with a 5% reduction in risk for sepsis (Zou et al., 2025). Circadian disruption was a key pathological feature exacerbating ferroptosis in SIC. A study has shown that ferroptosis increases over time in the LPS-induced H9C2 cell sepsis model, and this time-dependent ferroptosis was due to LPS-induced suppression of basic helix-loop-helix ARNT like 1 (BMAL1). Functional experiments confirmed that BMAL1 inhibited ferroptosis in H9C2 cells. Further studies revealed that BMAL1 inhibited ferroptosis by activating AKT and suppressing P53. In summary, BMAL1 inhibited LPS-induced ferroptosis in H9C2 cells through the AKT/P53 signaling pathway. Although further studies are needed, the findings of this study may provide new insights into the pathogenesis of SIC (Lin H. et al., 2023).

## 5 Therapeutic targets of Chinese herbal medicine in ferroptosis of sepsis-induced cardiomyopathy

The pathogenesis of SIC is complex, which poses a huge challenge to single-target therapeutic strategies. Against this backdrop, Chinese herbal medicine, with its unique advantages of multi-component synergy and multi-target regulation, has demonstrated potential application value in the treatment of SIC targeting the ferroptosis pathway (Ji et al., 2025; Fan et al., 2020).

### 5.1 Single herbal components

#### 5.1.1 Quercetin

Quercetin (QUE) is a naturally occurring flavonoid that is widely found in a variety of plants, which exhibits extensive biological activities such as antioxidant, anti-inflammation (Aghababaei and Hadidi, 2023; Yamaura et al., 2023). QUE conferred dual-pathway protection against ferroptosis in SIC. Clinically, SIC patients showed decreased serum GPX4 and silent information regulator 1 (SIRT1) levels, accompanied by elevated inflammatory cytokines and myocardial injury markers. In LPS-treated H9C2 cardiomyocytes, 80 µM QUE enhanced viability and suppressed ferroptosis *via* SIRT1/P53/SLC7A11 axis, while *in vivo* it dose-dependently alleviated cardiac injury, inflammation, and ferroptosis in SIC rats (Lin X. et al., 2023). In addition, QUE protected against LPS-induced myocardial injury by modulating the arachidonate 5-lipoxygenase (ALOX5)/phosphatidylinositol 3-kinase (PI3K)/AKT pathway. ALOX5, a key mediator of lipid peroxidation and ferroptosis, was upregulated in both SIC patients and LPS-treated AC16 cardiomyocytes. QUE downregulated ALOX5 expression, thereby activating the PI3K/AKT pathway. Functional studies confirmed that ALOX5 knockdown mimics QUE’s protective effect while ALOX5 overexpression reversed QUE’s inhibitory effects *in vitro* (Guan et al., 2024).

Together, these findings suggest that QUE alleviates SIC through multi-target regulation of ferroptosis. However, interpretation of these findings requires caution, as QUE belongs to pan-assay interference compounds (PAINS). Due to its redox activity and potential for nonspecific interactions, positive results from *in vitro* experiments may not always reflect target-specific pharmacological effects (Bolz et al., 2021). Therefore, greater weight should be given to evidence from animal studies, and further clinical validation is warranted.

#### 5.1.2 Matrine

Matrine, a natural alkaloid derived from *Sophora flavescens* Aiton (Zhang et al., 2020; Lin et al., 2022). In the LPS-induced SIC mouse model, matrine could significantly improve cardiac function and myocardial morphological abnormalities, with the best effect when intervened at a dose of 25 mg/kg. Network pharmacology analyses identified that matrine’s protective potential in SIC was closely associated with ferroptosis. Matrine could alleviate ferroptosis in SIC by up-regulating the expressions of GPX4 and SOD and down-regulating the expressions of ROS, MDA and ACSL4. Further study has shown that matrine could upregulate the levels of p-PI3K and p-AKT while the total levels of PI3K and AKT remained unchanged. In conclusion, matrine may inhibit ferroptosis by targeting the PI3K/AKT pathway, thereby alleviating SIC (Xiao et al., 2023).

#### 5.1.3 Alpinia officinarum hance extract

In LPS-induced sepsis mouse models, the aqueous extract of *Alpinia officinarum* Hance (AOE) exhibited dose-dependent cardioprotective effects. AOE administered at doses of 25, 50, and 100 mg/kg effectively ameliorated myocardial structural damage and reduced the expression levels of myocardial injury markers. On the one hand, AOE inhibited ferroptosis by stabilizing the intracellular Fe^2+^ levels, up-regulating the expression of GPX4 and down-regulating the expression of ACSL4. On the other hand, it could inhibit excessive inflammation by reducing the secretion of inflammatory factors. Notably, AOE could downregulate the expression of lncRNA myocardial infarction associated transcript (MIAT), positively regulate TNF receptor associated factor 6 (TRAF6) expression and inhibit the activation of nuclear factor kappa-B (NF-κB) pathway, which has been shown to be a key link in mediating inflammation and ferroptosis in sepsis. Furthermore, MIAT overexpression could reverse the cardiac protective effect of AOE, which further confirmed that lncRNA MIAT/TRAF6/NF-κB axis was the key target of AOE in suppressing ferroptosis and inflammation in SIC (Shi et al., 2024).

#### 5.1.4 Narciclasine

Narciclasine (Narc), a pancratistatin-type isocarbostyril alkaloid obtained from bulbs of the genus *Narcissus* (Amaryllidaceae), exhibited significant therapeutic potential in SIC by targeting ferroptosis and mitochondrial homeostasis (Liao et al., 2022). In both LPS and CLP sepsis models, administration of Narc could exert a dose-dependent cardioprotective effect and increase the 72-h survival rate. Narc significantly improved the profile of ferroptosis in SIC, specifically manifested as reducing iron overload, lowering MDA levels, alleviating GSH depletion, and up-regulating the expression of GPX4 and HO-1. Meanwhile, Narc enhanced mitochondrial quality control by promoting BCL2-interacting protein 3 (BNIP3)-dependent mitochondrial autophagy, thereby stabilizing mitochondrial membrane potential and improving mitochondrial respiratory function. Network pharmacology analysis and functional experiments confirmed that BNIP3 was the key target of Narc. *In vitro* and *in vivo* experiments, BNIP3 knockdown attenuated the anti-ferroptosis and pro-mitophagy effects of Narc, highlighting its core role in maintaining the integrity of mitochondrial structure and mediating the dual protective mechanism of Narc (Tang et al., 2025).

#### 5.1.5 Tectorigenin

Tectorigenin, a natural isoflavone derived from the rhizomes of *Iris tectorum* Maxim. (Iridaceae) and the roots of *Glycyrrhiza uralensis* Fisch. ex DC. (Fabaceae), has demonstrated significant cardioprotective effects in SIC (Rong et al., 2023). In the LPS-induced sepsis mouse model, tectorigenin pretreatment effectively mitigated myocardial dysfunction, reduced myocardial fiber structure damage and inhibited excessive inflammatory response. Tectorigenin inhibited ferroptosis of SIC by reducing intracellular iron accumulation, decreasing the expression of ACSL4, and increasing the expression of GPX4. Meanwhile, tectorigenin could alleviate myocardial inflammatory damage. It is worth noting that western blot and immunofluorescence experiments confirmed that tectorigenin exerted an anti-ferroptosis effect by specifically inhibiting the expression of SMAD family member 3 (SMAD3).

SMAD3 is a key target in the regulation of ferroptosis (Li, 2022; Kim et al., 2020). It was upregulated in SIC, which might aggravate myocardial injury by activating ferroptosis. The inhibition of SMAD3 by tectorigenin blocked this pathological cascade reaction, thereby reducing iron-dependent lipid peroxidation and myocardial injury. In summary, tectorigenin alleviateed SIC by inhibiting SMAD3-mediated ferroptosis and reducing myocardial inflammation (Fang et al., 2023).

#### 5.1.6 Wogonin

Wogonin, a natural flavonoid derived from the root of *Scutellaria baicalensis* Georgi (Lamiaceae). Pretreatment of mice with wogonin at doses of 20, 40, and 60 mg/kg showed a dose-dependent protective effect on the heart in the CLP-induced mice model. Mechanistically, wogonin could directly bind to ALOX15 and inhibit its activity. As a member of the lipoxygenase family, ALOX15 initiates the ferroptosis process by catalyzing the oxidation of PUFAs. Moreover, treatment with ML351, a specific inhibitor of ALOX15, mimicked the protective effect of Wogonin and significantly reduced myocardial ferroptosis. *In vitro* studies further confirmed that in LPS-stimulated HL-1 cardiomyocytes, wogonin blocked ferroptosis by inhibiting ALOX15 activity. This inhibitory effect on ferroptosis could be eliminated by the overexpression of ALOX15 or the supplementation of its downstream metabolite, 15-hydroxypereicosapentaenoic acid. In summary, wogonin exerted a protective effect on SIC by directly targeting the ferroptosis pathway mediated by ALOX15 (Ye et al., 2025).

#### 5.1.7 Resveratrol

Resveratrol (RSV), a natural polyphenolic compound found in the roots of *Polygonum cuspidatum* Siebold and Zucc. (Polygonaceae) and the fruits of *Vitis vinifera* L. (Vitaceae), exhibitsed cardioprotective effects in SIC by targeting ferroptosis through multiple regulatory pathways (Ren et al., 2021; Breuss et al., 2019). In CLP-induced sepsis rat models, the myocardial tissue expression of SIRT1 and NRF2 was downregulated. Both SIRT1 and NRF2 were key molecules for maintaining cellular redox homeostasis and regulating the process of ferroptosis. RSV administration (10, 30, 50 mg/kg) improved heart function and alleviated ferroptosis in a dose-dependent manner, with high-dose RSV mimicking the protective effects of Fer-1. Mechanistic validation using the SIRT1 inhibitor EX527 indicated that RSV inhibited ferroptosis by upregulating the SIRT1/NRF2 signaling pathway, as EX527 abolished RSV’s therapeutic effects (Zeng et al., 2023). RSV also inhibited cardiomyocyte ferroptosis by regulating the miR-149/high mobility group box 1 (HMGB1) axis in LPS-induced sepsis *in vitro* and *in vivo* models. Mechanically, RSV could upregulate the expression of miR-149, and miR-149 downregulated the HMGB1, which could promote ferroptosis. Further experiments confirmed that overexpression of HMGB1 could reverse the inhibitory effect of miR-149 on LPS-induced cardiac ferroptosis (Wang et al., 2022).

In conclusion, RSV inhibited ferroptosis by activating the SIRT/NRF2 signaling pathway and regulating the miR-149/HMGB1 axis.

#### 5.1.8 Baicalein

Baicalein, a natural flavonoid isolated from the root of *S. baicalensis* Georgi (Lamiaceae) (Dinda, 2017; Tan et al., 2022). Network pharmacology and experimental verification have confirmed that HIF1-α was the key mediator for baicalin to exert its inhibitory effect on ferroptosis. Mechanistic studies revealed that LPS downregulated miR-299b-5p in myocardial tissues, while baicalein administration reversed this reduction. Experiments confirmed direct targeting of HIF1-α by miR-299b-5p in cardiomyocytes, establishing a miR-299b-5p/HIF1-α axis. By upregulating miR-299b-5p, baicalein suppressed HIF1-α-mediated ferroptosis, thereby alleviating LPS-induced SIC (Wy, 2025).

#### 5.1.9 Puerarin

Puerarin, a major isoflavone found in the root of *Pueraria montana* var. *lobata* (Willd.) Maesen and S.M.Almeida ex Sanjappa and Predeep (Fabaceae), protected against SIC by activating adenosine 5′-monophosphate (AMP)-activated protein kinase (AMPK) to suppress ferroptosis. In LPS-treated rats, it improved cardiac function and reduced histological damage, concomitant with decreased serum injury markers (cTnI, CK-MB), pro-inflammatory cytokines (TNF-α, IL-6), and MDA. Mechanistically, puerarin upregulated GPX4 and ferritin while downregulating ACSL4, TFR1, and Fe^2+^. Crucially, these effects were AMPK-dependent: AMPK inhibitor Compound C reduced the P-AMPK/T-AMPK ratio, with corresponding changes in ferroptosis-related proteins and iron content. These findings confirmed that puerarin protected against SIC through regulating AMPK-mediated ferroptosis, offering a targeted approach against SIC (Zhou et al., 2022).

#### 5.1.10 Cyclovirobuxine D

Cyclovirobuxine D (CVB-D), a steroidal alkaloid isolated from *Buxus microphylla* Siebold and Zucc. (Buxaceae), was demonstrated therapeutic efficacy against SIC. Experimental studies have confirmed that CVB-D effectively activated the NRF2 signaling axis, thereby upregulating the expression of SLC7A11 and GPX4. Importantly, the NRF2-specific inhibitor ML385 could significantly weaken the induction effect of CVB-D on SLC7A11 and GPX4, which indicated the cardioprotective effect of NRF2 (Wang et al., 2023).

Together, these single herbal components target diverse ferroptosis-related mechanisms, including iron metabolism, lipid peroxidation, and antioxidant systems. These findings underscore the therapeutic potential of single herbal components in SIC by targeting ferroptosis through multi-target, pathway-specific regulation, providing a strong foundation for developing novel treatments for SIC.

### 5.2 Chinese herbal formulas

#### 5.2.1 TaoHeChengQi decoction

TaoHeChengQi decoction (THCQD) is a classical TCM formula composed of five medicinal herbs: *Prunus persica* (L.) Batsch (Rosaceae; Tao Ren), *Rheum palmatum* L. (Polygonaceae; Da Huang), *Cinnamomum cassia* (L.) J. Presl (Lauraceae; Gui Zhi), *Mirabilite* (Na_2_SO_4_; Mang Xiao), and *G. uralensis* Fisch. ex DC. (Fabaceae; Gan Cao). It was shown to inhibit myocardial ferroptosis in SIC both *in vivo* and *in vitro*. In CLP-induced sepsis mice models, THCQD was administered at doses of 2 and 4 g/kg as freeze-dried powder, which could reduce the levels of ferroptosis markers and restore ferroptosis-related protein expression. Based on body surface area conversion, these doses correspond to human equivalent doses of approximately 68.1 and 136.2 g crude herb per day for a 60 kg adult. Although these doses are higher than the conventional clinical dosage of THCQD, no apparent toxicity was observed in this study, and the high doses were primarily employed to elucidate the underlying mechanisms. Network pharmacology and cellular thermal shift assay identified NRF2 as a critical target, with western blotting and immunofluorescence confirming THCQD-mediated NRF2 activation in cardiac tissues. Functional validation using the NRF2 inhibitor ML385 or NRF2 knockout mice showed partial reversal of THCQD’s anti-ferroptosis effects, demonstrating that THCQD suppressed myocardial ferroptosis *via* NRF2 activation (Lu et al., 2024).

Using the HPLC-Q-Exactive-MC analysis, a total of 72 compounds were identified. Among them, protocatechuic acid, gallic acid, catechin, caffeic acid, ferulic acid and quercetin have been shown to exert anti-ferroptotic effects by activating the NRF2 signaling pathway in various diseases, including metabolic dysfunction-associated fatty liver disease (Feng et al., 2024), intervertebral disc degeneration (Zhu et al., 2025), atherosclerosis (Guo et al., 2025), cerebral ischemia injury (Li et al., 2024), and cisplatin-induced cardiotoxicity (Wang et al., 2022). This suggests these compounds might be the key active components of THCQD that exert anti-ferroptosis effects. However, it remains unclear which specific compounds in THCQD activate NRF2 to exert protective effects in SIC. The anti-ferroptosis effect of THCQD might also be the result of the combined effect of multiple active components rather than being caused by a single compound.

#### 5.2.2 YiQiFuMai injection

YiQiFuMai injection (YQFM), a modern preparation derived from the classical traditional Chinese formula Sheng Mai San, showed therapeutic efficacy against SIC by targeting myocardial ferroptosis through the SLC7A11/GPX4 signaling axis. This formulation consists of three medicinal components: the root of *Panax ginseng* C.A.Mey. (Araliaceae; Renshen), the tuberous root of *Ophiopogon japonicus* (L.f.) Ker Gawl. (Asparagaceae; Maidong), and the fruit of *Schisandra chinensis* (Turcz.) Baill. (Schisandraceae; Wuweizi). Functional experiments with GPX4 inhibitor showed that blocking GPX4 activity abolished the anti-ferroptosis effects of YQFM, including its effects on reducing lipid peroxidation and enhancing cardiomyocyte viability (Guo et al., 2024). According to relevant literature reports, the main active components ginsenoside Rg1 and ophiopogonin D in YQFM have anti-ferroptosis effects (Pu et al., 2025; Lin et al., 2024). However, there is currently a lack of direct evidence to identify which specific components in YQFM exert anti-ferroptosis effects by regulating the SLC7A11/GPX4 axis.

It is worth noting that in this study, the rat dosages were 0.23, 0.45, and 0.9 mg/kg. Based on body surface area conversion, when calculated for a 60 kg adult body weight, the daily dosages were 2.4, 4.2, and 9.0 mg, respectively. Clinically, the recommended clinical dosage is 5.2 g per day. Therefore, the human equivalent dosage used in this study was much lower than the routine clinical dosage. This suggests that the dosage used in this study has good safety in clinical applications, and it also indicates that the anti-ferroptosis effect observed within this dosage range may also exist or even be stronger at the routine clinical dosage.

## 6 Discussion

### 6.1 Ferroptosis as a central mechanism in sepsis-induced cardiomyopathy

SIC is a severe complication of sepsis, and its pathogenesis is complex, involving various pathological processes. In recent years, the crucial role of ferroptosis in SIC has become increasingly clear. This review elucidates the molecular mechanisms of ferroptosis in SIC and summarizes the research progress on the cardioprotective effects of Chinese herbal medicine monomers and formulas targeting ferroptosis.

Iron metabolism disorder is the key initiating step that triggers ferroptosis during SIC. Although the circulating iron level decreases during sepsis, there is an abnormal accumulation of iron in the myocardial tissue. This contradictory phenomenon is mainly achieved through the following pathways: upregulation of TFR1 expression mediates excessive iron uptake by cardiomyocytes; NCOA4-mediated ferritinophagy promotes intracellular iron release; LCN2 mediates an increase in Fe^2+^ of LIP; and upregulation of HO-1 accelerates heme degradation and releases a large amount of iron. These factors collectively contribute to myocardial iron overload, ultimately leading to uncontrolled lipid peroxidation. Further studies have shown that the lipid peroxidation process of ferroptosis in SIC is regulated by multiple signaling pathways, including ICA69-STING, m^6^A RNA methylation modification, and circadian rhythm disruption.

The pathogenesis of SIC involves multiple forms of cell death types. In addition to ferroptosis, apoptosis, pyroptosis, and necroptosis also play significant roles. These various forms of cell death types do not exist independently but are interrelated. For instance, the activation of the inflammasome can simultaneously trigger pyroptosis and ferroptosis, while the receptor-interacting protein kinase family members mediate the crosstalk between ferroptosis and necroptosis. Caspase is not only the executor of apoptosis but also participates in the regulation of pyroptosis and necroptosis. GSDMD is not only the core effector molecule of pyroptosis but can also interact with necroptosis (Wu et al., 2025; Sheng et al., 2023). However, at present, the research on the interaction between TCM regulation of ferroptosis and other forms of cell death types in SIC is still relatively limited.

### 6.2 Therapeutic potential of Chinese herbal medicine targeting ferroptosis and limitation of current experimental evidence

Chinese herbal medicine exhibits substantial potential in mitigating SIC by targeting ferroptosis through multi-level regulation. Table 1 summarizes the main findings of the mechanisms and targets related to the effects of Chinese herbal medicine on ferroptosis in SIC. However, some of the studies in Table 1 have limitations: Firstly, some studies adopted pre-treatment administration protocol. Although this approach helps to reveal the underlying mechanism, it differs from the actual clinical situation. Clinically, patients with sepsis are already in a disease state when they seek medical attention, and treatment intervention is usually initiated after the onset of sepsis. Secondly, among the studies cited in this review, some utilized the CLP-induced sepsis models, while others employed the LPS-induced sepsis model. The LPS-induced sepsis model has the advantages of simple operation, good repeatability and short modeling time. However, LPS is only a component of the cell wall of gram-negative bacteria and can only simulate the pathological process of gram-negative bacterial endotoxemia. The inflammatory response induced by LPS is single and fails to cover the diversity of bacteria, the formation of infection foci, and the dynamic evolution of immune response, which is different from the complex pathological features of human sepsis. In contrast, the CLP model is regarded as the “gold standard” for studying sepsis (Dejager et al., 2011; Seemann et al., 2017). Therefore, when interpreting the research results based on the LPS model, the limitations of it should be fully considered. Future research should prioritize the use of the CLP model and verify key findings in multiple models. Thirdly, among the studies included in this review, researchers seemed tend to validate the well-recognized NRF2/SLC7A11/GPX4 axis, which serve as a key defense system against ferroptosis. However, the NRF2/SLC7A11/GPX4 axis is one of the important pathways through which these TCM exert their anti-ferroptosis effects, but it is not the sole mechanism.

**TABLE 1:** Studies on cardioprotective Chinese herbal medicines against ferroptosis in sepsis-induced cardiomyopathy.

Author	Chinese herbal medicine	Experimental	Model	Administration	Dosage	Target	Signaling pathway	Refs
Xin Lin	Quercetin	*In vitro* *In vivo*	H9C2 cells CLP-Rats	Incubation Injection	20, 40, 80, 160 μmol/L 20, 40 mg/kg	GPX4, GSH, MDA, SLC7A11, PTGS2	SIRT1/P53	Lin X. et al. (2023)
Fang Guan	Quercetin	*In vitro*	AC16 cell	Incubation	15, 30, 45 μmol/L	ROS, GSH, GPX4, ALOX5	PI3K/AKT	Guan et al. (2024)
Yuhong Xiao	Matrine	*In vivo*	LPS-Mice	Intraperitoneal injection	25 mg/kg	ROS, MDA, SOD GPX4, ACSL4	PI3K/AKT	Xiao et al. (2023)
Yao Shi	Alpinia officinarum Hance Extract	*In vivo*	LPS-Mice	Intraperitoneal injection	25, 50, 100 mg/kg	MDA, SOD, GPX4, ACSL4	LncRNA MIAT/TRAF6/NF-κB	Shi et al. (2024)
Rong Tang	Narciclasine	*In vivo* *In vitro*	LPS-Mice CLP-Mice NRCMs	Intraperitoneal injection Incubation	5, 20 mg/kg 30, 100, 300 nmol/L	GSH, ROS, TFR1 MDA, GPX4 HO-1 SLC7A11, FSP1 ACSL4	-	Tang et al. (2025)
Xiaowei Fang	Tectorigenin	*In vivo*	LPS-Mice	Intraperitoneal injection	2.5, 5, 10 mg/kg	ACSL4, GPX4, SOD, MDA.	-	Fang et al. (2023)
Hua Ye	Wogonin	*In vivo* *In vitro*	CLP-Mice HL-1 cells	Intraperitoneal injection Incubation	20, 40, 60 mg/kg 10 μmol/L	ALOX5, GPX4, PTGS2, MDA 4-HNE, GSH/GSSG.	-	Ye et al. (2025)
Youcheng Zeng	Resveratrol	*In vivo* *In vitro*	CLP-Rats H9C2 cells	Intraperitoneal injection Incubation	10, 30, 50 mg/kg 1, 10, 25, 50, 75, 100 μmol/L	ACSL4, GPX4, MDA.	SIRT1/NRF2	Zeng et al. (2023)
Xiaoli Wang	Resveratrol	*In vivo*	LPS-mice	Intraperitoneal Injection	50 mg/kg	GSH, ROS, MDA.	miR-149/HMGB1	Wang et al. (2022)
WenYan Zhou	Baicalein	*In vivo* *In vitro*	LPS-mice H9C2 cells	Intravenous Injection	1, 5, 10 mg/kg 5, 10, 20 μmol/L	GPX4, MDA.	miR-299b-5p/HIF1-α	Wy (2025)
Bin Zhou	Puerarin	*In vivo*	LPS-Rats	Intraperitoneal injection	100 mg/kg	GPX4, ACSL4, TFR1, ferritin. GSH, SOD, MDA.	AMPK	Zhou et al. (2022)
Jianxin Wang	Cyclovirobuxine D	*In vivo* *In vitro*	CLP-Rats H9C2 cells	Gavage Incubation	4 mg/kg 10 mmol/L	GSH, SOD, MDA, GPX4, PTGS2, SLC7A11, FTH, TFR1	NRF2	Wang et al. (2023)
SiMin Lu	TaoHe ChengQi decoction	*In vivo* *In vitro*	CLP-Mice NRCMs	Gavage Incubation	2, 4 g/kg 50, 100, 200 μg/mL	ACSL4, GSH 4-HNE, MDA, GPX4, TFR1, HO-1, NQO1	NRF2	Lu et al. (2024)
Liying Guo	YiQiFuMai Injection	*In vivo* *In vitro*	CLP-Rats H9C2 cells	Intraperitoneal injection	0.23, 0.45, 0.9 mg/kg 0.05, 0.1, 0.5, 1, 2 mg/mL	GPX4, GSH/GSSG, SLC7A11, ROS SLC3A2, MDA 4-HNE.	-	Guo et al. (2024)

Furthermore, although targeting ferroptosis may be a therapeutic strategy for SIC, systematic inhibition of ferroptosis may compromise the host’s defense mechanisms, especially the nutritional immune function conferred by hypoferremia (Ganz and Nemeth, 2024). Hypoferremia is an adaptive response under infection, which inhibits the growth and reproduction of pathogens and enhances the host’s ability to resist infection by reducing the concentration of serum iron (Nairz and Weiss, 2020; Marchetti et al., 2020). This process plays an important defensive role in sepsis. Although inhibiting ferroptosis may further reduce the serum iron concentration and enhance the inhibitory effect of nutritional immunity to some extent, long-term or excessive intervention may lead to iron deficiency, which can impair immune cells’ function, thereby weakening overall host defense.

### 6.3 Evidence hierarchy and quality assessment of current studies

The quality of included animal studies was evaluated using the SYRCLE risk-of-bias tool (Supplementary Table S1). Evidence hierarchy and appraisal of all included studies were summarized in Supplementary Table S2. Although studies showed low risk of loss to follow-up and reporting bias, random sequences generation, baseline characteristics, random housing, detection bias were frequently unclear, indicating potential methodological limitations.

Among the 14 studies included in this review, only one study incorporated human observational data, whereas no clinical trials specifically evaluating ferroptosis-targeted TCM interventions in SIC were identified. 8 studies combined *in vitro* and *in vivo* experiments, 5 relied exclusively on animal models, and 1 study was limited to *in vitro* experiments. Therefore, the current evidence primarily supports mechanistic plausibility and preclinical efficacy rather than clinical effectiveness. Importantly, several studies were conducted exclusively in LPS-induced models, and some flavonoid compounds (e.g., QUE and baicalein) may be susceptible to PAINS-related concerns, highlighting the need for rigorous pharmacological validation and clinical confirmation. Therefore, the overall certainty of evidence remains limited, and findings from *in vitro* experiments should be interpreted with greater caution than those from animal experiments.

## 7 Conclusion and perspectives

Ferroptosis emerges as an important mechanism in SIC, driven by intricate interactions between iron metabolism dysregulation, uncontrolled lipid peroxidation, epigenetic modifications, and circadian rhythm disruption. Emerging evidence indicates that Chinese herbal medicines and their bioactive constituents exert cardioprotective effects by modulating multiple ferroptosis-related pathways, highlighting ferroptosis as a promising therapeutic target in SIC.

However, several challenges remain. First, the current evidence is based on *in vitro* and *in vivo* experiments, and high quality clinical evidence is lacking. In the future, well-designed clinical studies should be conducted to improve translational relevance; Second, the precise molecular crosstalk between ferroptosis and other cell death types in SIC requires deeper exploration. The “key targets” in the cell death pathway should be explored to develop multi-pathway synergistic regulatory drugs. Third, the components of TCM formulas are complex. Therefore, it is necessary to systematically identify their active ingredients, clarify the mechanism of synergy, and conduct comprehensive pharmacokinetic studies, so as to more clearly understand their bioavailability and the process of their action in the body. Fourth, in future explorations of ferroptosis inhibition strategies for SIC treatment, it is essential to balance the cardioprotective effects with the impact on host defense mechanisms. Optimizing the intervention methods, such as adopting ferroptosis inhibition strategies targeting cardiomyocytes, may minimize systemic iron disruption, ensuring the safety and effectiveness of the treatment.

Overall, ferroptosis represents a promising but still evolving therapeutic target in SIC. Additional pharmacological validation, rigorous mechanistic investigations, and high quality clinical studies are required before ferroptosis-targeted interventions can be translated into clinical practice.

## Glossary

SIC: Sepsis-induced cardiomyopathy
TCM: Traditional Chinese Medicine
HO-1: heme oxygenase 1
SLC7A11: solute carrier family 7 member 11
HIF1-α: hypoxia inducible factor 1 subunit alpha
P53: tumor protein p53
Fer-1: ferrostatin-1
GPX4: glutathione peroxidase 4
PUFAs: polyunsaturated fatty acids
MDA: malondialdehyde
4-HNE: 4-hydroxynonenal
AI: anemia of inflammation
IL-6: interleukin-6
SOFA: sequential organ failure assessment
CK-MB: creatine kinase MB isoenzymes
cTnI: cardiac troponin I
NCOA4: nuclear receptor coactivator 4
TFR1: transferrin receptor 1
LPS: lipopolysaccharide
LCN2: lipocalin 2
FPN1: ferroportin 1
PTGS2: prostaglandin-endoperoxide synthase 2
ACSL4: acyl-CoA synthetase long chain family member 4
GSH: glutathione
SOD: superoxide dismutase
CLP: cecal ligation and puncture
ICA69: islet cell autoantigen 69
STING: stimulator of interferon genes
FUNDC1: FUN14 domain containing 1
FTO: fat mass and obesity-associated protein
BACH1: BTB and CNC homology 1
METTL3: methyltransferase 3
YTHDF2: YTH N6-methyladenosine RNA binding protein F2
METTL5: methyltransferase 5
NRF2: nuclear factor erythroid 2-related factor 2
BMAL1: basic helix-loop-helix ARNT like 1
p-AKT: phosphorylated protein kinase B
QUE: quercetin
SIRT1: sirtuin 1
ALOX5: 5-lipoxygenase
PI3K: phosphatidylinositol 3-kinase
AKT: protein kinase B
AOE: alpinia officinarum hance extract
MIAT: myocardial infarction associated transcript
TRAF6: TNF receptor associated factor 6
NF-κB: nuclear factor kappa-B
Narc: narciclasine
BNIP3: BCL2 interacting protein 3
SMAD3: SMAD family member 3
ALOX15: 15-lipoxygenase
RSV: resveratrol
HMGB1: high mobility group box 1
AMP: adenosine 5‘-monophosphate
AMPK: adenosine 5‘-monophosphate-activated protein kinase
CVB-D: cyclovirobuxine D
THCQD: TaoHe ChengQi decoction
NRCMs: neonatal rat cardiomyocytes
YQFM: YiQiFuMai injection
PAINS: pan-assay interference compounds
