# Supplementary material for: Targeting ferroptosis in sepsis-induced cardiomyopathy: mechanisms and therapeutic potential of traditional Chinese Medicine
Source: Front Pharmacol. 2026 Aug 21;17:1785167. doi: 10.3389/fphar.2026.1785167 (PMC13543871; doi:10.3389/fphar.2026.1785167)
Supplement: Supplementary file 1 [file Supplementaryfile1.docx]

**Supplementary Table 1. Evaluation results of the SYRCLE animal experiment risk assessment included in this review**

| Quality assessment  References | Selection bias | | | Performance bias | | Detection bias | | Loss to follow-up bias | Reporting Bias | Other Bias |
| --- | --- | --- | --- | --- | --- | --- | --- | --- | --- | --- |
|  | Random sequences generation | Baseline characteristics | Allocation concealment | Random housing | Blinding | Random outcome assessment | Blinding | Incomplete outcomes  data | Selecting report | Bias from other resources |
| Xin Lin^[68]^ | uncertain | uncertain | uncertain | low risk | uncertain | uncertain | uncertain | low risk | low risk | low risk |
| Yuhong Xiao^[72]^ | uncertain | uncertain | uncertain | low risk | uncertain | uncertain | uncertain | low risk | low risk | low risk |
| Yao Shi^[73]^ | uncertain | uncertain | uncertain | low risk | uncertain | uncertain | uncertain | low risk | low risk | low risk |
| Rong Tang^[75]^ | uncertain | uncertain | uncertain | low risk | uncertain | uncertain | uncertain | low risk | low risk | low risk |
| Xiaowei Fang^[79]^ | uncertain | uncertain | uncertain | low risk | uncertain | uncertain | uncertain | low risk | low risk | low risk |
| Hua Ye^[80]^ | uncertain | uncertain | uncertain | low risk | uncertain | uncertain | uncertain | low risk | low risk | low risk |
| Youchen Zeng^[83]^ | uncertain | uncertain | uncertain | low risk | uncertain | uncertain | uncertain | low risk | low risk | low risk |
| Supplementary Table 1 (continued) | | | | | | | | | | |
| Quality assessment  References | Selection bias | | | Performance bias | | Detection bias | | Loss to follow-up bias | Reporting Bias | Other Bias |
|  | Random sequences generation | Baseline characteristics | Allocation concealment | Random housing | Blinding | Random outcome assessment | Blinding | Incomplete outcomes  data | Selecting report | Bias from other resources |
| Xiaoli^[84]^ Wang | uncertain | uncertain | uncertain | low risk | uncertain | uncertain | uncertain | low risk | low risk | low risk |
| Wenyan Zhou^[87]^ | uncertain | uncertain | uncertain | low risk | uncertain | uncertain | uncertain | low risk | low risk | low risk |
| Bin Zhou^[88]^ | uncertain | uncertain | uncertain | low risk | uncertain | uncertain | uncertain | low risk | low risk | low risk |
| Jianxin Wang^[89]^ | uncertain | uncertain | uncertain | low risk | uncertain | uncertain | uncertain | low risk | low risk | low risk |
| Simin Lu^[90]^ | uncertain | uncertain | uncertain | low risk | uncertain | uncertain | uncertain | low risk | low risk | low risk |

| Supplementary Table 1 (continued) | | | | | | | | | | |
| --- | --- | --- | --- | --- | --- | --- | --- | --- | --- | --- |
| Quality assessment  References | Selection bias | | | Performance bias | | Detection bias | | Loss to follow-up bias | Reporting Bias | Other Bias |
|  | Random sequences generation | Baseline characteristics | Allocation concealment | Random housing | Blinding | Random outcome assessment | Blinding | Incomplete outcomes  data | Selecting report | Bias from other resources |
| Liying Guo^[96]^ | uncertain | uncertain | uncertain | low risk | uncertain | uncertain | uncertain | low risk | low risk | low risk |

**Supplementary Table 2. Evidence hierarchy and appraisal of included studies**

| **Chinese Herbal Medicine** | **Evidence Hierarchy** | **Strengths** | **Key Limitations** | **Reference** |
| --- | --- | --- | --- | --- |
| Quercetin | Clinical observation + In vivo + In vitro | Multi-level evidence; patient biomarkers combined with H9C2 and CLP-rat validation; multiple ferroptosis markers assessed | No clinical intervention data; limited pharmacokinetic evidence; focused mainly on SIRT1/P53 pathway | 68 |
| Quercetin | In vitro only | Detailed mechanistic study; ALOX5 gain/loss of function validation | No animal or clinical validation; potential PAINS-related interference; translational relevance uncertain | 69 |
| Matrine | In vivo only | Evaluated oxidative stress and ferroptosis markers | No clinical evidence; LPS model only; no validation experiment | 73 |
| Alpinia officinarum Hance Extract | In vivo only | Dose-dependent effects | No clinical evidence; crude extract; active compounds undefined; LPS model only | 74 |
| Narciclasine | In vivo + In vitro | LPS and CLP models; dose-dependent effects; comprehensive assessment; | No clinical evidence; lack of pharmacokinetic and safety studies | 76 |

Supplementary Table 2 (continued)

| **Chinese Herbal Medicine** | **Evidence Hierarchy** | **Strengths** | **Key Limitations** | **Reference** |
| --- | --- | --- | --- | --- |
| Tectorigenin | In vivo only | Dose-dependent effects | No clinical evidence; LPS model only; limited mechanistic investigation; tectorigenin pretreatment | 80 |
| Wogonin | In vivo + In vitro | CLP model; dose-dependent effects; evaluated lipid peroxidation and ALOX5-related ferroptosis | No clinical evidence; target specificity not fully validated; Wogonin pretreatment | 81 |
| Resveratrol | In vivo + In vitro | CLP model; Mechanistic validation; dose-dependent effects; with ferroptosis inhibitor as control | No clinical evidence; pleiotropic effects make target attribution difficult | 84 |
| Resveratrol | In vivo only | Investigated upstream miRNA-mediated regulation | No clinical evidence; LPS model only; limited ferroptosis-specific validation | 85 |
| Baicalein | In vivo + In vitro | Cellular and animal validation; explored miRNA/HIF-1α pathway; with ferroptosis inhibitor as control | No clinical evidence; LPS model only; flavonoid-related PAINS concerns cannot be excluded | 88 |

Supplementary Table 2 (continued)

| **Chinese Herbal Medicine** | **Evidence Hierarchy** | **Strengths** | **Key Limitations** | **Reference** |
| --- | --- | --- | --- | --- |
| Puerarin | In vivo only | Evaluated both ferroptosis and iron metabolism markers; with ferroptosis activator as control | No clinical evidence; LPS model only; no mechanistic cellular validation | 89 |
| Cyclovirobuxine D | In vivo + In vitro | CLP model; NRF2-mediated ferroptosis regulation investigated; dose-dependent effects; | No clinical evidence; pharmacokinetic data unavailable; no mechanistic validation | 90 |
| TaoHe ChengQi decoction | In vivo + In vitro | Clinically used preparation; CLP model and primary cardiomyocytes; broad ferroptosis marker panel | No SIC clinical trial; complex composition; active ingredients and direct targets remain unclear | 91 |
| YiQiFuMai Injection | In vivo + In vitro | Clinically used preparation; multiple ferroptosis markers assessed; dose-dependent effects; with ferroptosis activator and inhibitor as control | No SIC clinical trial; mechanistic depth limited; complex composition; active ingredients and direct targets remain unclear | 97 |
